# Age-Associated Neurological Complications of COVID-19: A Systematic Review and Meta-Analysis

**DOI:** 10.3389/fnagi.2021.653694

**Published:** 2021-08-02

**Authors:** Brianne N. Sullivan, Tracy Fischer

**Affiliations:** ^1^Department of Microbiology and Immunology, Tulane University School of Medicine, New Orleans, LA, United States; ^2^Neuroscience Program, Tulane Brain Institute, School of Science and Engineering, Tulane University, New Orleans, LA, United States; ^3^Division of Comparative Pathology, Tulane National Primate Research Center, Covington, LA, United States; ^4^Tulane Brain Institute, Tulane University School of Medicine, New Orleans, LA, United States

**Keywords:** brain, COVID-19, cerebrovascular events, SARS-CoV-2, encephalopathy, aging brain

## Abstract

The outbreak of the novel and highly infectious severe acute respiratory syndrome coronavirus-2 (SARS-CoV-2) has resulted in hundreds of millions of infections and millions of deaths globally. Infected individuals that progress to coronavirus disease-19 (COVID-19) experience upper and lower respiratory complications that range in severity and may lead to wide-spread inflammation and generalized hypoxia or hypoxemia that impacts multiple organ systems, including the central and peripheral nervous systems. Since the SARS-CoV-2 outbreak, multiple reports continue to emerge that detail neurological symptoms, ranging from relatively mild (e.g., impaired taste and/or smell) to severe (e.g., stroke), suggesting SARS-CoV-2 may be neurotropic and/or contribute to nervous system injury through direct and/or indirect mechanisms. To gain insight into the types of neurological complications associated with SARS-CoV-2 infection and their possible relationship with age, sex, COVID-19 severity, and comorbidities, we performed a systematic review of case reports and series published in 2020 – April 4, 2021 of infected patients with neurological manifestations. Meta-analyses were conducted using individual patient data from reports where these data could be extracted. Here, we report neurological injury occurs across the lifespan in the context of infection, with and without known comorbidities, and with all disease severities, including asymptomatic patients. Older individuals, however, are more susceptible to developing life-threatening COVID-19 and cerebrovascular disease (CVD), such as stroke. A mild but inverse correlation with age was seen with CNS inflammatory diseases, such as encephalitis, as well as taste and/or smell disorders. When reported, increased age was also associated with comorbid cardiovascular risk factors, including hypertension, diabetes mellitus, and lipid disorders, but not with obesity. Obesity did correlate with development of critical COVID-19. Discussion into potential pathophysiological mechanisms by which neurological symptoms arise and long-term consequences of infection to the nervous system is also provided.

## Introduction

Infectious disease, ranging in severity from symptoms of a mild cold to severe acute respiratory distress are attributed to coronaviruses (CoV)s. The majority of this large family of viruses are transmitted among non-human species, however, occasional zoonosis has resulted in seven known CoV strains that infect and cause disease in humans. Of these, three human CoVs (huCoVs) strains have emerged over the past two decades that can promote severe disease and even death. Severe acute respiratory coronavirus (SARS-CoV) and Middle East respiratory syndrome (MERS)-CoV, emerged in 2003 and 2012, respectively, causing significant global illness and mortality (Drosten et al., 2003; Memish et al., 2013). In December 2019, a novel CoV strain, now designated SARS-CoV-2, was first reported to infected humans and cause severe disease, termed CoV disease-19 (COVID-19). While most individuals with COVID-19 experience mild to moderate symptoms, others develop more severe disease, leading to death in a subset of these patients. Rapid transmission of the virus has resulted in a global pandemic resulting in hundreds of millions of infections and millions of deaths, worldwide, that remains on-going at the time of this review (Huang et al., 2020; WHO, 2021).

Although primarily considered a virus impacting the respiratory system, an increasing number of case studies have highlighted substantial neurological consequences of SARS-CoV-2 infection. Indeed, the Centers for Disease Control (CDC) lists new confusion or the inability to arouse as indicators of severe COVID-19 presentation, necessitating emergency medical attention (CDC, 2020). Early reports from Wuhan, China alerted the neuroinvasive potential of SARS-CoV-2, as multiple patients developed headache and dizziness, anosmia, and/or ageusia, which were often reported as initial symptoms of infection and disease (Chen N. et al., 2020; Huang et al., 2020; Mao et al., 2020; Yang et al., 2020). In addition, acute onset of more serious neurological symptoms, including altered mental status (encephalopathy), meningoencephalitis, demyelinating diseases, and stroke are increasingly reported in SARS-CoV-2 infected patients (Al-Olama et al., 2020; Farhadian et al., 2020; Lodigiani et al., 2020; Lu et al., 2020; Scullen et al., 2020; TunÇ et al., 2020). Many reports that reveal the age of the subjects studied suggest that patients older than 50 years are more likely to experience severe neurological complications, however, varying new onset neurological manifestations have also been reported among younger individuals and appear to be a common complication of COVID-19. As such, there is a critical need for investigating the impact of COVID-19 on the central nervous system (CNS). Here, we present evidence for a direct or indirect role of SARS-CoV-2 in promoting neurological disease in individuals across the lifespan via a systematic review of the literature and meta-analyses. We also discuss potential pathophysiology of SARS-CoV-2-associated CNS injury and the potential for long-term neurological complications of infection in recovered patients, including the potential impact of disease on pathological brain aging.

## Methods

### Search Strategy and Study Selections

A systematic review was conducted for the purposes of identifying the population of COVID-19 patients diagnosed with new onset neurological condition(s) during the disease course. This review was designed and organized in accordance with the Preferred Reporting Items for Systematic Reviews (PRISMA) guidelines (Moher et al., 2009). The database PubMed-NCBI was systematically searched for peer-reviewed literature presenting original clinical data of COVID-19 patients diagnosed with a neurological condition. The search of PubMed-NCBI alone is considered comprehensive and reliable, as over 90% of MEDLINE is covered by this database, thus the search of additional databases was deemed unnecessary (Williamson and Minter, 2019). Manuscripts published from 2019 to April 4, 2021 were interrogated using the following search terms: (“COVID-19” OR “SARS-CoV-2”) AND (“Brain” OR “Neuro” OR “Stroke” OR “Seizure” OR “Anosmia” OR “Ageusia” OR “Guillain-Barré” OR “Headache” OR “Dizziness” OR “Confusion” OR “Impaired Consciousness” OR “Seizure” OR “Encephalopathy” OR “Meningitis”) NOT “review.” The search was restricted to full text peer-reviewed reports available in English containing original clinical data. Preprint articles were not included. The purpose of this systematic review and meta-analysis was to assess the type and incidence of neurological complications of COVID-19 in relation to age. As such, only published articles with original clinical data containing the following criteria were included: (1) age of patient(s) featured in the study, (2) a diagnosis of new onset neurological manifestations, and (3) laboratory-confirmed SARS-CoV-2 infection. Exclusion criteria included: (1) any known pre-existing neurological conditions, (2) known co-current viral or parasitic infection, and/or (3) opinions, viewpoints, personal anecdotes, and reviews. Seizure was reported in a SARS-CoV-2 positive 6-week-old male (Dugue et al., 2020), however, this case was excluded from analysis because a history of seizure could not be ruled out, due to the young age. An 80-year-old woman with Alzheimer’s dementia, who developed stroke (Xiong et al., 2020) and a 52-year-old HIV-infected woman with posterior reversible encephalitic syndrome (PRES) (Anand et al., 2020a) were also excluded from analyses, as these comorbidities could not be ruled-out as significant confounders to the development of neurological disease in the context of COVID-19.

### Data Extraction and Synthesis

Articles vetted for inclusion were independently reviewed by BNS and TF, and the following information was extracted for analysis: age, gender, neurological manifestation, COVID-19 symptom severity, comorbidities, and presence of virus in CSF or autopsied brain. All data were captured and maintained in a Microsoft Excel workbook. Any disagreement regarding inclusion was resolved by discussion.

To reduce the effects of heterogeneity among the case reports, neurological diagnoses/symptoms were evaluated and categorized as cerebrovascular disease (CVD), peripheral neuropathy, encephalopathy, demyelinating disease, smell and/or taste disorder, and CNS inflammatory disease. The category “other” was included to capture patients who exhibited neurological symptoms, but the underlying cause was not determined or identified. This is expanded in Supplementary Table 1. Dichotomous outcomes were created for each category of CNS disease for statistical tests. Reported comorbidities were also reduced to dummy variables for assessing potential relationships with hypertension (HTN), diabetes mellitus (DM), lipid disorder, obesity, none, and other. This is expanded in Supplementary Table 2. For analyses, comorbidities were scaled based on their overall relationship with CVD, which had the strongest association with age and COVID-19 severity. Scores were designed as follows: None = 0, other = 1, obesity = 2, lipid disorder = 3, DM = 4, HTN = 5. This allowed for the inclusion of multiple reported comorbidities by synthesizing a “comorbidity score” for each patient equal to the sum of the individual scores. For example, a patient with HTN and DM would have a comorbidity score = 9. “None” includes only reports that specifically stated no comorbidities. Reports that did not include comorbidities were excluded from analyses that required these data. COVID-19 severity was converted to ordinal variables as follows: asymptomatic = 0, mild = 1, moderate = 2, severe = 3, critical = 4.

### Statistical Analyses

Statistical tests were performed using Prism 9 for MacOS (v9.1.1) and the on-line statistics software, Intellectus Statistics (2021)^1^. Graphs were constructed with Prism and Microsoft Excel. Summary statistics for individual patient data were calculated for each variable. A general assessment of the relationship between age and each neurological disease category was conducted through simple linear regression using dummy coding (dichotomous outcomes) for the neurological disease category. Pearson correlation matrices were constructed to assess pair-wise relationships between variables. Cohen’s standard was used to evaluate the strength of the relationships, where coefficients between ±0.10 and ±0.29 represent a small effect size, coefficients between ±0.30 and ±0.49 represent a moderate effect size, and coefficients of ±0.50 and above indicate a large effect size (Cohen, 1998). The result of the correlations was examined using Holm corrections to adjust for multiple comparisons based on α = 0.05. Analysis of variance (ANOVA) was conducted to assess if there were significant differences in COVID-19 severity or comorbidities score between the levels of neurological disease category. Tukey pairwise comparisons were conducted for all significant effects based on α = 0.05. For each statistical test, only data from patients with all variables investigated were included. Cases with missing data were excluded from analysis. As such, the *n* for each test or summary is reported.

## Results

### Search Results and Population Characteristics

**FIGURE 1:**
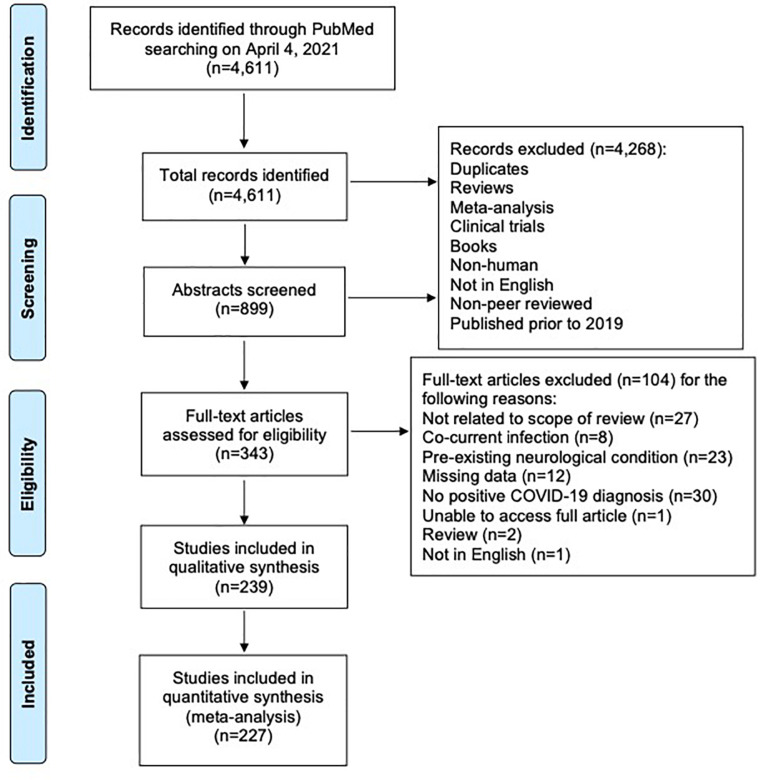
PRISMA flow diagram of systematic literature search and screening for studies of COVID-19 patients with neurological conditions.

**FIGURE 2:**
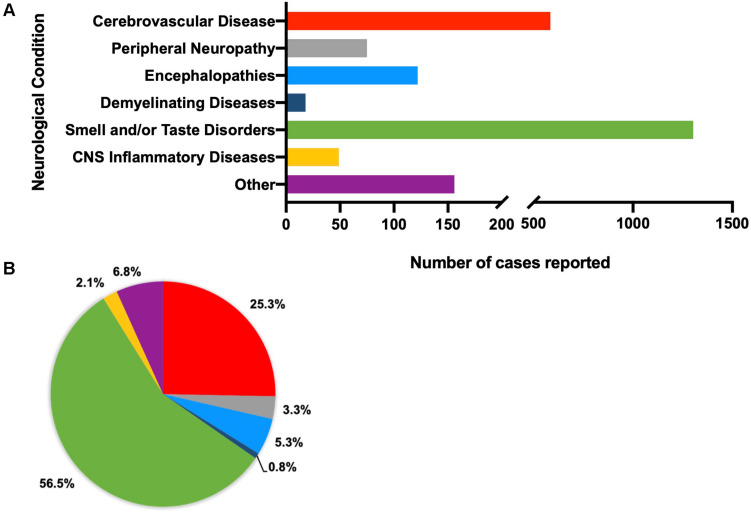
Total number **(A)** and percent of **(B)** reported neurological conditions occurring in patients, regardless of demographics (total *n* = 2,390). Individual diagnoses have been categorized as cerebrovascular diseases (*n* = 592), peripheral neuropathies (*n* = 75), encephalopathies (*n* = 175), demyelinating diseases (*n* = 23), smell and/or taste disorders (*n* = 1,303), CNS inflammatory diseases (*n* = 45), and other (neurological symptoms that cannot be attributed to a specific neurological condition, such as headache, seizure, ataxia, aphasia) (*n* = 177).

Of an initial 4,611 records retrieved, 4,372 were excluded as per our exclusion criteria (Figure 1). A total of 239 articles were included in an overall assessment for the prevalence of neurological conditions reported in all patients with confirmed COVID-19 (*n* = 2,307) (Figure 2). The prevalence of COVID-19 patients with neurological conditions were assessed by age (years) from a total of 230 articles (*n* = 584) in which individual ages could be extracted (Table 1). Patient diagnoses were categorized under the following general neurological conditions: cerebrovascular disease (CVD), peripheral neuropathies, encephalopathies, demyelinating diseases, smell and/or taste disorders, and CNS inflammatory diseases. An additional category of “other” was ascribed to patients who exhibited neurological symptoms, but the underlying cause was not determined or identified (Figure 2). Smell and/or taste disorders were the most prevalent neurological manifestation with 1,303 cases identified, or 56.5% of the total. CVD, including stroke and microhemorrhages, was seen less frequently, but impacted approximately one quarter of the total (*n* = 584). Each of the remaining neurological conditions comprised less than ten percent of the total reported with encephalopathy and “other” accounting for 5.3% (*n* = 122) and 6.8% (*n* = 156), respectively. Less prevalent, but nonetheless significant neurological conditions also reported include peripheral neuropathy [e.g., Guillain-Barré Syndrome (GBS) and critical illness neuromyopathy (CIM)], CNS inflammatory disease (e.g., encephalitis and myelitis), and demyelinating disease [e.g., multifocal demyelinating lesions and acute disseminated encephalomyelitis (ADEM)], constituting a respective 3.3% (*n* = 75), 2.1% (*n* = 49), and 0.8% (*n* = 18) of the total subjects.

**TABLE 1:** Total number (*n* = 584) and percent of each neurological condition reported in patients diagnosed with COVID-19 per decade of age.

Age range	≤ 9	10–19	20–29	30–39	40–49	50–59	60–69	70–79	≥ 80

#Cases reported	7	22	19	60	72	100	154	121	29

Neurological diagnosis	*N* (%) [reference]								
**Cerebrovascular diseases**	**2 (28.6)** (Xiong et al., 2020; Tiwari et al., 2021)	**3 (13.6)** (Asif and O’ Mahony, 2020; Gulko et al., 2020; Dakay et al., 2021)	**3 (15.8)** (Cavalcanti et al., 2020; de Sousa et al., 2020; Dakay et al., 2021)	**19 (31.7)** (Al Saiegh et al., 2020; Cavalcanti et al., 2020; Cezar-Junior et al., 2020; Chia et al., 2020; Diaz-Segarra et al., 2020; Fara et al., 2020; Kantonen et al., 2020; Oxley et al., 2020; Pisano et al., 2020; Trifan et al., 2020a, b; Vu et al., 2020; Wang et al., 2020; Al-Mufti et al., 2021; Sabayan et al., 2021; Shah et al., 2021)	**31 (43.1)** (Agarwal et al., 2020; Anzalone et al., 2020; Cavalcanti et al., 2020; de Almeida Lima et al., 2020; Fabbri et al., 2021; Frisullo et al., 2020; Gupta et al., 2020; Heman-Ackah et al., 2020; Lapergue et al., 2020; Nicholson et al., 2020; Oxley et al., 2020; Patel et al., 2020; Sabayan et al., 2021; Toledano-Massiah et al., 2020; Trifan et al., 2020a; TunÇ et al., 2020; Wang et al., 2020; Yaghi et al., 2020; Al-Mufti et al., 2021; Fu et al., 2021; Mullaguri et al., 2021)	**46 (46.0)** (Avci et al., 2020; Beyrouti et al., 2020; Cezar-Junior et al., 2020; D’Anna et al., 2020; Diaz-Segarra et al., 2020; Fabbri et al., 2021; Fara et al., 2020; Fayed et al., 2020; Heman-Ackah et al., 2020; Jaunmuktane et al., 2020; Kremer et al., 2020a; Lapergue et al., 2020; Mansour et al., 2020; Morassi et al., 2020; Motoie et al., 2020; Nepal et al., 2020; Nicholson et al., 2020; Panico et al., 2020; Sangalli et al., 2020; Sugiyama et al., 2020; Toledano-Massiah et al., 2020; Trifan et al., 2020a; Wang et al., 2020; Xiong et al., 2020; Yaghi et al., 2020; Al-Mufti et al., 2021; Fu et al., 2021; Harrogate et al., 2021; Mousa-Ibrahim et al., 2021; Mullaguri et al., 2021; Prasad et al., 2021; Sabayan et al., 2021)	**70 (45.5)** (Al Saiegh et al., 2020; Anand et al., 2020a; Azpiazu Landa et al., 2020; Beyrouti et al., 2020; Bigliardi et al., 2020; Bolaji et al., 2020; Cannac et al., 2020; Cezar-Junior et al., 2020; Co et al., 2020; D’Anna et al., 2020; Díaz-Pérez et al., 2020; Diaz-Segarra et al., 2020; Guillan et al., 2020; Gulko et al., 2020; Hemasian and Ansari, 2020; Imoto et al., 2020; Jaunmuktane et al., 2020; Kantonen et al., 2020; Kremer et al., 2020a; Lapergue et al., 2020; Morassi et al., 2020; Nicholson et al., 2020; Roy-Gash et al., 2020; Saitta et al., 2020; Shawkat et al., 2020; Shoskes et al., 2020; Siepmann et al., 2021; Soldatelli et al., 2020; Trifan et al., 2020a; TunÇ et al., 2020; Vattoth et al., 2020; Wang et al., 2020; Xiong et al., 2020; Yaghi et al., 2020; Yong et al., 2020; Al-Mufti et al., 2021; Fabbri et al., 2021; Garví López et al., 2021; Khan et al., 2021; Mousa-Ibrahim et al., 2021; Sabayan et al., 2021)	**69 (57.0)** (Agarwal et al., 2020; Al-Dalahmah et al., 2020; Avula et al., 2020; Azpiazu Landa et al., 2020; Beyrouti et al., 2020; Burkert and Patil, 2020; Cezar-Junior et al., 2020; D’Anna et al., 2020; Fara et al., 2020; Fayed et al., 2020; Gonçalves et al., 2020; Kremer et al., 2020a; Mohamed et al., 2020; Morassi et al., 2020; Papi et al., 2020; Sangalli et al., 2020; Trifan et al., 2020a; TunÇ et al., 2020; Viguier et al., 2020; Xiong et al., 2020; Yaghi et al., 2020; Zayet et al., 2020; Al-Mufti et al., 2021; Fabbri et al., 2021; Harrogate et al., 2021; Mousa-Ibrahim et al., 2021; Mullaguri et al., 2021; Robles, 2021; Sabayan et al., 2021)	**21 (72.4)** (Alay et al., 2020; Avula et al., 2020; Beyrouti et al., 2020; D’Anna et al., 2020; Gonçalves et al., 2020; Kantonen et al., 2020; Kremer et al., 2020a; Xiong et al., 2020; Zayet et al., 2020; Al-Mufti et al., 2021; Sabayan et al., 2021; Siepmann et al., 2021)

**Peripheral neuropathies**		**2 (9.1)** (Khalifa et al., 2020; Manji et al., 2020)	**2 (10.5)** (Hutchins et al., 2020; Toscano et al., 2020)	**6 (10.0)** (Ameer et al., 2020; Dinkin et al., 2020; Gutiérrez-Ortiz et al., 2020; Homma et al., 2020; Lantos et al., 2020; Rajdev et al., 2020)	**8 (11.1)** (Bigaut et al., 2020; Khaja et al., 2020; Liberatore et al., 2020; Tiet and AlShaikh, 2020; Velayos Galán et al., 2020; Abolmaali et al., 2021; Manganotti et al., 2021; Nanda et al., 2021)	**15 (15.0)** (Assini et al., 2020; Barrachina-Esteve et al., 2020; Chan et al., 2020; Gale et al., 2020; Gutiérrez-Ortiz et al., 2020; Hirayama et al., 2020; Korem et al., 2020; Manganotti et al., 2020; Naddaf et al., 2020; Petrelli et al., 2020; Rana et al., 2020; Sancho-Saldaña et al., 2020; Toscano et al., 2020; Webb et al., 2020; Abolmaali et al., 2021; Nanda et al., 2021)	**12 (7.8)** (Abrams et al., 2020; Assini et al., 2020; Bracaglia et al., 2020; Juliao Caamaño and Alonso Beato, 2020; Sedaghat and Karimi, 2020; Senel et al., 2020; Tankisi et al., 2020; Toscano et al., 2020; Wada et al., 2020; Zhao et al., 2020; Bueso et al., 2021; Nasuelli et al., 2021)	**16 (13.2)** (Alberti et al., 2020; Bigaut et al., 2020; Coen et al., 2020; Dinkin et al., 2020; Fernández-Domínguez et al., 2020; Marta-Enguita et al., 2020; Su et al., 2020; Toscano et al., 2020; Manganotti et al., 2021; Nanda et al., 2021; Nasuelli et al., 2021)	**2 (6.9)** (Abolmaali et al., 2021; Manganotti et al., 2021)

**Encephalopathies**	**2 (28.6)** (Abel et al., 2020; De Paulis et al., 2020)		**2 (10.5)** (Agarwal et al., 2020; Babar et al., 2020)	**7 (11.7)** (Benameur et al., 2020; Dakay et al., 2020; Kamal et al., 2020; Kantonen et al., 2020; Pascual-Goñi et al., 2020; Radnis et al., 2020; Zayet et al., 2021)	**9 (12.5)** (Fischer et al., 2020; Franceschi et al., 2020; Hosseini et al., 2020; Muccioli et al., 2020; Ordoñez-Boschetti et al., 2020; Radnis et al., 2020; Scullen et al., 2020; Edén et al., 2021; Matos et al., 2021)	**11 (10.0)** (Abenza-Abildúa et al., 2020; Anand et al., 2020a; Anzalone et al., 2020; Chaumont et al., 2020b; Dixon et al., 2020; Naaraayan et al., 2020; Nicolas-Jilwan and Almaghrabi, 2020; Priftis et al., 2020; Scullen et al., 2020; Harrogate et al., 2021; Perrin et al., 2021)	**25 (16.2)** (Benameur et al., 2020; Chaumont et al., 2020b; Delorme et al., 2020; Díaz-Pérez et al., 2020; Franceschi et al., 2020; Kakadia et al., 2020; Kantonen et al., 2020; Pascual-Goñi et al., 2020; Princiotta Cariddi et al., 2020; Radnis et al., 2020; Roy-Gash et al., 2020; Scullen et al., 2020; Shoskes et al., 2020; Vaschetto et al., 2020; Yong et al., 2020; Edén et al., 2021; Garví López et al., 2021; Perrin et al., 2021; Zayet et al., 2021)	**14 (1.6)** (Alkeridy et al., 2020; Balestrino et al., 2020; Butt et al., 2020; Cebrián et al., 2020; Chaumont et al., 2020b; Delorme et al., 2020; Farhadian et al., 2020; Hosseini et al., 2020; Morassi et al., 2020; Soysal and Kara, 2020; Edén et al., 2021; Harrogate et al., 2021; Mullaguri et al., 2021; Perrin et al., 2021)	**2 (6.9)** (Kantonen et al., 2020; Edén et al., 2021)

**Demyelinating diseases**		**1 (4.5)** (de Miranda Henriques-Souza et al., 2021)		**2 (3.3)** (Agarwal et al., 2020; McCuddy et al., 2020)	**2 (2.8)** (Lopes et al., 2020; Pessoa Neto et al., 2020)	**7 (7.0)** (Abdi et al., 2020; Brun et al., 2020; Langley et al., 2020; Lopes et al., 2020; McCuddy et al., 2020; Parsons et al., 2020; Chang et al., 2021)	**4 (2.6)** (Jaunmuktane et al., 2020; Kakadia et al., 2020; Wada et al., 2020; Khan et al., 2021)	**3 (2.5)** (Cani et al., 2021; McCuddy et al., 2020; Reichard et al., 2020)	

**Smell and/or Taste disorder**		**8 (36.4)** (Chiu et al., 2005; Erdede et al., 2020; Hatipoglu et al., 2020; Mak et al., 2020)	**5 (26.3)** (Babar et al., 2020; Laurendon et al., 2020; Pissurno et al., 2020; Politi et al., 2020; Smith et al., 2020)	**9 (15.0)** (Faber et al., 2020; Gilani et al., 2020; Homma et al., 2020; Vargas-Gandica et al., 2020)	**8 (11.1)** (Bigaut et al., 2020; Gane et al., 2020; Gilani et al., 2020; Sampaio Rocha-Filho and Voss, 2020; Vargas-Gandica et al., 2020; Manganotti et al., 2021; Matos et al., 2021)	**4 (4.0)** (Melley et al., 2020; Panico et al., 2020; Vargas-Gandica et al., 2020)	**10 (6.5)** (Chow et al., 2020; de Oliveira et al., 2020; Hjelmesæth and Skaare, 2020; Jacob et al., 2020; Vargas-Gandica et al., 2020; Bueso et al., 2021; Edén et al., 2021)	**4 (3.3)** (Bigaut et al., 2020; Manganotti et al., 2021)	**2 (6.9)** (Hjelmesæth and Skaare, 2020; Vargas-Gandica et al., 2020)

**CNS inflammatory diseases**	**3 (42.9)** (Kaur et al., 2020; Raj et al., 2020; Kim et al., 2021)	**4 (4.5)** (Lin et al., 2020; Natarajan et al., 2020; Bektaş et al., 2021)	**3 (15.8)** (Elkhaled et al., 2020; Laurendon et al., 2020; Moriguchi et al., 2020)	**7 (11.7)** (Álvarez Bravo et al., 2020; Benameur et al., 2020; Efe et al., 2020; Handa et al., 2020; Fumery et al., 2021; Povlow and Auerbach, 2021)	**5 (6.9)** (Agarwal et al., 2020; Ghosh et al., 2020; Heller et al., 2020; Kadono et al., 2020; Fabbri et al., 2021)	**13 (13.0)** (Águila-Gordo et al., 2020; Kremer et al., 2020a; Monti et al., 2020; Picod et al., 2020; Poyiadji et al., 2020; Fabbri et al., 2021)	**18 (11.7)** (Chaumont et al., 2020a; Chow et al., 2020; Hanafi et al., 2020; Kremer et al., 2020a; Munz et al., 2020; Sotoca and Rodríguez-Álvarez, 2020; Vaschetto et al., 2020; Fabbri et al., 2021)	**6 (5.0)** (Kremer et al., 2020a; Fabbri et al., 2021)	**1 (3.4)** (Kremer et al., 2020a)

**Other**		**4 (18.2)** (Hatipoglu et al., 2020; Seth and Kushwaha, 2020)	**4 (21.1)** (Anand et al., 2020b; Lyons et al., 2020; Silva et al., 2020)	**10 (16.7)** (Dinkin et al., 2020; Kantonen et al., 2020; Noro et al., 2020; Pascual-Goñi et al., 2020; Silva et al., 2020; Tu et al., 2020)	**9 (12.5)** (Anand et al., 2020b; Sampaio Rocha-Filho and Voss, 2020; Silva et al., 2020; Fabbri et al., 2021)	**4 (4.0)** (Fasano et al., 2020; Ortiz-Seller et al., 2020; Fabbri et al., 2021)	**15 (9.7)** (Anand et al., 2020b; de Oliveira et al., 2020; Jacob et al., 2020; Kantonen et al., 2020; Le Guennec et al., 2020; Ottaviani et al., 2020; Pascual-Goñi et al., 2020; Silva et al., 2020; Fabbri et al., 2021)	**9 (7.4)** (Anand et al., 2020b; Dinkin et al., 2020; Elgamasy et al., 2020; Logmin et al., 2020; Vollono et al., 2020; Fabbri et al., 2021)	**1 (3.4)** (Kantonen et al., 2020)

### Summary of Individual Patient Data for Meta-Analyses

Data of 510 patients were extracted from 227 published reports, from which age and neurological complication could be matched. When possible, sex, COVID-19 severity, comorbidities, and the presence of detectable virus in cerebrospinal fluid (CSF) were also captured. As described above, neurological diagnoses were broadly categorized under CVD, CNS inflammatory disease, demyelinating disease, encephalopathy, peripheral neuropathy, taste/smell disorders, and other. Frequencies and percentages of individual diagnoses included under these categories are listed in Supplementary Table 1. The most frequently reported CVD was stroke of various types (*n* = 230, 89%). Among CNS inflammatory diseases, meningoencephalitis was most frequently observed (*n* = 22, 47%). Acute disseminated encephalomyelitis (ADEM) was the most frequently reported demyelinating disease (*n* = 9, 60%). Within the category of encephalopathy, a diagnosis of various types of encephalopathy were reported with the highest frequency (*n* = 47, 83%), while different manifestations of GBS were the most frequently observed peripheral neuropathy (*n* = 54, 84%). Loss of smell (anosmia) was the most frequent complication of taste/smell disorders (*n* = 11, 31%) and various manifestations of headache (*n* = 18, 49%) were the most frequently reported neurological manifestation categorized as “other,” which includes neurological manifestations for which the underlying cause was not identified.

Comorbidities were reported for 363 of the 510 patients for which individual patient data could be extracted. Risk factors for cardiovascular disease, including hypertension (HTN), diabetes mellitus (DM), obesity, and hyperlipidemia, were the most frequent comorbidities among COVID-19 patients with neurological manifestations (Supplementary Table 2). To assess the association of the stated comorbidities with neurological manifestations, comorbidities were limited to HTN, DM, obesity, and lipid disorders, which includes hyperlipidemia, dyslipidemia, and hypercholesterolemia. All other reported comorbidities were included as “other.”

Although the number of males outnumbered females, the mean age did not differ significantly between the two sexes, or from the mean age of a cohort of individuals for which sex was not specified (Table 2). Summary statistics for individual patient data were calculated for each variable, and frequencies and percentages were split by sex and reported in Table 3. Frequencies and percentages were only calculated on available data (n/a = not available excluded). This is reflected in the *n* reported for each variable. The most frequently observed neurological disease category for all patients regardless of sex, specified or not, was cerebrovascular disease (*n* = 257, 50% of total). Moderate COVID-19 severity was most often reported for all male and female subjects, however, patients for which sex was not specified (*n* = 57), severe COVID-19 was most frequently reported. Although no comorbidities (none) appear to be most frequently reported among all subjects (*n* = 131, 36%), when taken together, HTN, with and without additional comorbidities, is most frequently reported *in toto* (*n* = 165, 45%), as well as separately among males (*n* = 96, 46%) and persons for which sex was not specified (*n* = 27, 57%). No comorbidities (none) and HTN, with and without additional comorbidities, were equally reported among females (*n* = 42, 39%). CSF was assessed for detectable virus in 122 of the total 510 patients but only identified in four cases (Table 3), which included a 31-year-old male and a 74-year-old female with altered mental status (encephalopathy), a 24-year-old male with meningoencephalitis, and a 68-year-old male who developed stroke (Cebrián et al., 2020; Kamal et al., 2020; Moriguchi et al., 2020; Saitta et al., 2020).

**TABLE 2:** Summary statistics for age of patients *in toto* and by sex.

	Mean age				Min	Max
*Sex*	(years)	*SD*	*n*	*SE*_*M*_	(years)	(years)
*All subjects*	55.37	18.16	510	0.80	2	94
*Male*	55.55	17.75	278	1.06	2	94
*Female*	54.33	19.99	175	1.51	3	92
*Not specified*	57.67	13.74	57	1.82	31	93

**TABLE 3:** Frequencies and percentages of neurological disease, COVID-19 severity, comorbidities, and detectable virus in CSF by sex.

Variable	*n*	Female	Male	Not specified
***Neurological disease category***	**510**			
*Cerebrovascular disease*		68 (39%)	133 (48%)	56 (98%)
*CNS inflammatory disease*		20 (11%)	26 (9%)	0 (0%)
*Demyelinating disease*		6 (3%)	8 (3%)	1 (2%)
*Encephalopathy*		25 (14%)	32 (12%)	0 (0%)
*Taste/smell disorders*		21 (12%)	15 (5%)	0 (0%)
*Peripheral neuropathy*		19 (11%)	44 (16%)	0 (0%)
*Other*		16 (9%)	20 (7%)	0 (0%)
***COVID-19 severity***	**495**			
*Asymptomatic*		20 (12%)	16 (6%)	3 (5%)
*Mild*		21 (12%)	44 (16%)	2 (4%)
*Moderate*		61 (36%)	73 (27%)	10 (18%)
*Severe*		31 (18%)	67 (25%)	35 (61%)
*Critical*		37 (22%)	68 (25%)	7 (12%)
***Comorbidities (reclassified)***	**363**			
*None*		42 (39%)	78 (37%)	11 (23%)
*DM*		2 (2%)	17 (8%)	2 (4%)
*DM, lipid disorder*		1 (1%)	1 (0%)	2 (4%)
*DM, obesity*		1 (1%)	0 (0%)	0 (0%)
*DM, other*		1 (1%)	1 (0%)	1 (2%)
*HTN*		20 (19%)	27 (13%)	8 (17%)
*HTN, DM*		5 (5%)	24 (11%)	0 (0%)
*HTN, DM, lipid disorder*		1 (1%)	3 (1%)	5 (11%)
*HTN, DM, lipid disorder, other*		0 (0%)	1 (0%)	3 (6%)
*HTN, DM, obesity*		1 (1%)	4 (2%)	0 (0%)
*HTN, DM, obesity, lipid disorder, other*		1 (1%)	0 (0%)	0 (0%)
*HTN, DM, obesity, other*		1 (1%)	2 (1%)	0 (0%)
*HTN, DM, other*		2 (2%)	6 (3%)	0 (0%)
*HTN, lipid disorder*		1 (1%)	10 (5%)	5 (11%)
*HTN, lipid disorder, other*		3 (3%)	0 (0%)	2 (4%)
*HTN, obesity*		1 (1%)	4 (2%)	0 (0%)
*HTN, obesity, lipid disorder*		0 (0%)	1 (0%)	0 (0%)
*HTN, obesity, lipid disorder, other*		1 (1%)	0 (0%)	0 (0%)
*HTN, obesity, other*		0 (0%)	1 (0%)	0 (0%)
*HTN, other*		5 (5%)	13 (6%)	4 (9%)
*Lipid disorder*		1 (1%)	1 (0%)	3 (6%)
*Lipid disorder, other*		0 (0%)	1 (0%)	0 (0%)
*Obesity*		4 (4%)	5 (2%)	1 (2%)
*Obesity, other*		5 (5%)	0 (0%)	0 (0%)
*Other*		8 (7%)	9 (4%)	0 (0%)
***Virus in CSF***	**122**			
*No*		50 (98%)	67 (96%)	1 (100%)
*Yes*		1 (2%)	3 (4%)	0 (0%)

*Due to rounding errors, column wise percentages may not equal 100%. CNS, Central nervous system; DM, Diabetes mellitus; HTN, Hypertension; CSF, Cerebral spinal fluid.*

Finally, frequencies and percentages were calculated for neurological disease category split by COVID-19 severity (*n* = 495; Table 4). Regardless of disease severity, CVD was the most frequently observed category of neurological disease, which may reflect a more serious injury, such as stroke, being more likely to prompt a case report. It is important to note, that CVD was reported in the context of SARS-COV-2 infection among individuals with few or no other symptoms typically associated with COVID-19.

**TABLE 4:** Frequencies and percentages of observed neurological disease split by COVID-19 severity (*n* = 495).

*Neurological disease category*	Asymptomatic	Mild	Moderate	Severe	Critical
*Cerebrovascular disease*	18 (46%)	25 (37%)	55 (38%)	88 (66%)	58 (52%)
*CNS inflammatory disease*	2 (5%)	4 (6%)	15 (10%)	6 (5%)	17 (15%)
*Demyelinating disease*	1 (3%)	1 (1%)	1 (1%)	2 (2%)	10 (9%)
*Encephalopathy*	4 (10%)	7 (10%)	10 (7%)	24 (18%)	12 (11%)
*Loss of taste/smell*	7 (18%)	10 (15%)	16 (11%)	0 (0%)	3 (3%)
*Peripheral neuropathy*	5 (13%)	15 (22%)	19 (13%)	12 (9%)	12 (11%)
*Other*	2 (5%)	5 (7%)	28 (19%)	1 (1%)	0 (0%)

*Due to rounding errors, column wise percentages may not equal 100%.*

### Age-Associated Neurological Complications of COVID-19

**FIGURE 3:**
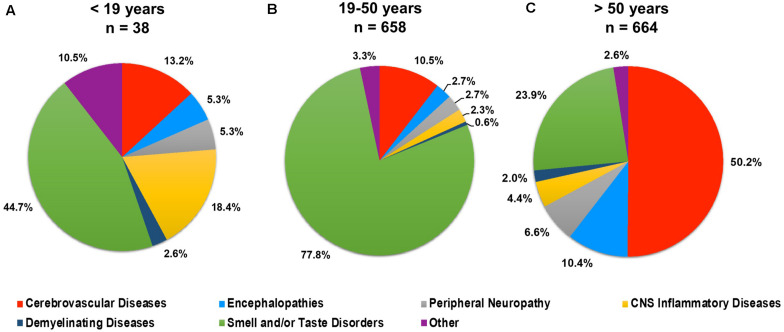
Percent of total neurological conditions (*n* = 1,360) occurring in **(A)** children (<19 years), **(B)** young adults (19–50 years), and **(C)** older adults (>50 years) diagnosed with COVID-19.

**FIGURE 4:**
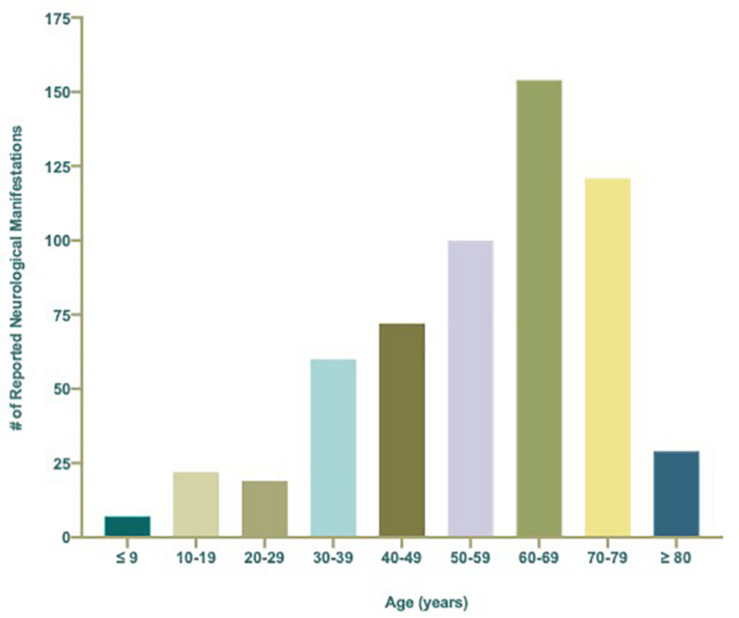
Total number (*n* = 584) of reported neurological manifestations occurring in patients with COVID-19 per decade ranging from ≤9 to ≥80 years of age.

Neurological conditions were evaluated by age, where the individual age or cohort age range was able to be determined and stratified to assess the overall frequency of specific types of neurological complications affecting children (<19 years), young adults (19–50 years), and older adults (>50) infected with SARS-CoV-2 (Figure 3). More specific details relating the number and percent of COVID-19 patients diagnosed with neurological conditions are stratified by decade of age and included in Table 1. Overall, patients 60–69 years showed the greatest population with neurological conditions (*n* = 154) and those less than or equal to 9 years of age had the least (*n* = 7) (Table 1 and Figure 4). Age and other available population characteristics of multicenter, retrospective, and observational studies with large cohorts reporting neurological conditions from which individual matched patient data could not be discerned is detailed in Supplementary Table 3. Instances where data were able to be extracted from these reports is detailed.

**FIGURE 5:**
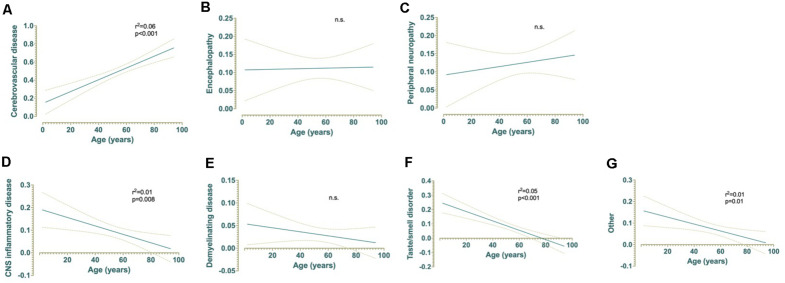
Simple linear regressions demonstrating the effect of age on **(A)** cerebrovascular disease, **(B)** encephalopathy, **(C)** peripheral neuropathy, **(D)** CNS inflammatory disease, **(E)** demyelinating disease, **(F)** taste and/or smell disorders, and **(G)** other non-specific neurological symptoms and their relationships with age (years) for patients with COVID-19 (*n* = 510).

Linear regression analyses were conducted to assess whether age significantly predicted any category of neurological complications of SARS-CoV-2 infection (Figure 5). The results of the linear regression model were significant for CVD, *F*(1,508) = 30.08, *p* < 0.001, *r*^2^ = 0.06, indicating that approximately 6% of the variance in CVD is explainable by increased age (Figure 5A). In contrast, taste/smell disorder was associated with decreased age, *F*(1,508) = 28.73, *p* < 0.001, *r*^2^ = 0.05, indicating that approximately 5% of the variance in this category is explainable by age (Figure 5F). A mild, but nonetheless statistically significant inverse relationship between age and CNS inflammatory disease or other was also observed. Approximately 1% of the variance in observation of COVID-19 patients with CNS inflammatory disease [*F*(1,508) = 7.19, *p* = 0.008, *r*^2^ = 0.01] or other [*F*(1,508) = 6.70, *p* = 0.01, *r*^2^ = 0.01] is also explainable by decreased age (Figures 5D,G). Age did not explain a significant proportion of variation in the observed frequencies of encephalopathy, peripheral neuropathy, or demyelinating disease (Figures 5B,C,E).

### Relationship of Age, COVID-19 Severity, and Comorbidities on Neurological Manifestations of COVID-19

**FIGURE 6:**
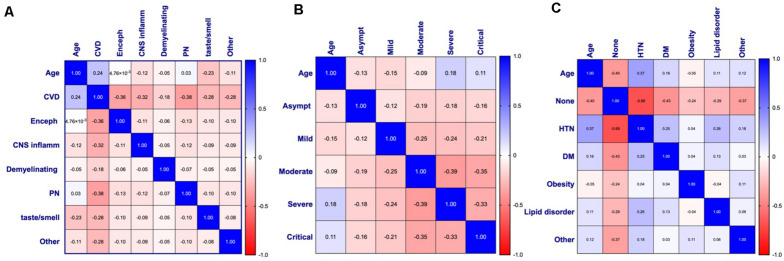
Pearson’s correlation matrices and heat maps for **(A)** age and neurological disease (*n* = 510), **(B)** age and COVID-19 symptom severity (*n* = 495), and **(C)** age and comorbidities (*n* = 363) of patients with COVID-19 and diagnosed with a neurological condition(s). Pearson’s correlation coefficients are displayed and assigned color based on the distance from zero, with blue representing positive and red representing negative correlations. CVD, Cerebrovascular disease; Enceph, Encephalopathy; CNS inflamm, Central nervous system inflammatory disease; PN, Peripheral neuropathy; Asympt, Asymptomatic; HTN, Hypertension; DM, Diabetes mellitus.

A Pearson correlation analysis was conducted among age, sex, each category of neurological disease, COVID-19 severity, and individual comorbid factors to assess the relationships among these variables and displayed as heat maps based on the coefficient between variables (Figure 6). Cases with incomplete data for the variables being assessed were excluded from analysis, resulting in a different *n* for each analysis.

In agreement with the linear regression analyses, a significant positive correlation between age and CVD was seen (*p* < 0.001) with a coefficient (*r*_p_) of 0.24, indicating a small effect size (Figure 6A). Small effect size was also observed with age and CNS inflammatory disease (*r*_p_ = −0.12, *p* = 0.008), smell and/or taste disorder (*r*_p_ = −0.23, *p* < 0.001), and other (*r*_p_ = −0.11, *p* = 0.01), all of which show a negative correlation with age (Figure 6A). The relationship between age and COVID-19 severity was statistically significant for all disease severities (Figure 6B). A negative relationship was observed between age and asymptomatic (*r*_p_ = −0.13, *p* = 0.005), mild (*r*_p_ = −0.15, *p* < 0.01), or moderate (*r*_p_ = −0.09, *p* = 0.046) COVID-19 severity, while a positive correlation was seen between age and severe (*r*_p_ = 0.18, *p* < 0.001) or critical (*r*_p_ = 0.11, *p* = 0.013) disease (Figure 6B).

Apart from obesity, the relationship between age and comorbidities was statistically significant for all types examined (Figure 6C). A moderate effect size (*r*_p_ = −0.40, *p* < 0.001) between age and stated no comorbidities (none) was observed, indicating that as age increases, the category of “none” tends to decrease. In contrast, the significant positive relationship between age and HTN (*r*_p_ = 0.37, *p* < 0.001), DM (0 *r*_p_ = 0.16, *p* = 0.002), or lipid disorders (*r*_p_ = 0.11, *p* = 0.042), suggests comorbid cardiovascular risk factors may contribute to the increased risk for CVD and/or severe-critical COVID-19 observed. Although the relationship between age and “other” was found to be statistically significant with a small effect size (*r*_p_ = 0.12, *p* = 0.023), the wide variety of conditions included in this category do not point to any one condition as being significant.

**FIGURE 7:**
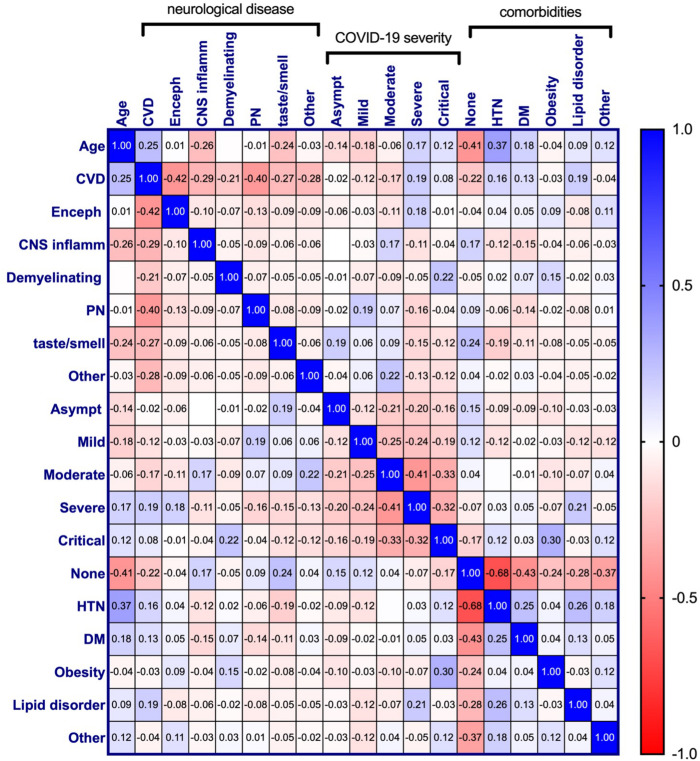
Pearson correlation matrix for age, neurological disease, COVID-19 symptom severity, and comorbidities of COVID-19 patients diagnosed with a neurological condition(s) (n = 303). Pearson’s correlation coefficients are displayed and assigned color based on the distance from zero, with blue representing positive and red representing negative correlations. CVD, Cerebrovascular disease; Enceph, Encephalopathy; CNS inflamm, Central nervous system inflammatory disease; PN, Peripheral neuropathy; Asympt, Asymptomatic; HTN, Hypertension; DM, Diabetes mellitus.

Relationships among all variables were also assessed and displayed in Figure 7. This revealed additional associations with neurological disease among patients for which all variables were available (*n* = 350). Age retained the strongest relationship with CVD, however, a significant positive correlation of CVD with HTN (*r*_p_ = 0.16, *p* = 0.002), DM (*r*_p_ = 0.13, *p* = 0.014), lipid disorders (*r*_p_ = 0.19, *p* < 0.001), and severe COVID-19 (*r*_p_ = 0.19, *p* < 0.001) were also seen. Encephalopathy correlated positively with severe COVID-19 (*r*_p_ = 0.18, *p* = 0.001) and was seen most frequently among individuals with comorbid conditions categorized as “other” (*r*_p_ = 0.11, *p* = 0.033). CNS inflammatory disease showed a positive correlation with moderate COVID-19 severity (*r*_p_ = 0.17, *p* = 0.002), as well as patients without comorbid disease (*r*_p_ = 0.17, *p* = 0.001). No significant relationship between comorbidities and demyelinating disease was observed, however, it did correlate with critical COVID-19 (*r*_p_ = 0.22, *p* < 0.001). These results, however, may be less reliable due to a low number of patients within this neurological disease category (*n* = 12). There appears to be a small positive relationship between demyelinating disease and obesity, however, this did not reach statistical significance. Like demyelinating disease, obesity had a positive association with critical COVID-19 (*r*_p_ = 0.30, *p* < 0.001). Peripheral neuropathy correlated with mild COVID-19 (*r*_p_ = 0.19, *p* < 0.001) but not with any comorbid condition. Interestingly, impaired taste/smell only reached significant positive associations with asymptomatic COVID-19 (*r*_p_ = 0.19, *p* < 0.001) and no comorbidities (*r*_p_ = 0.24, *p* < 0.001). Although the reason for this is unclear, in the absence of more critical symptoms, impairments in taste and/or smell may be more discernable by patients.

### Effect of COVID-19 Severity and Comorbidities on Neurological Disease Outcome

**FIGURE 8:**
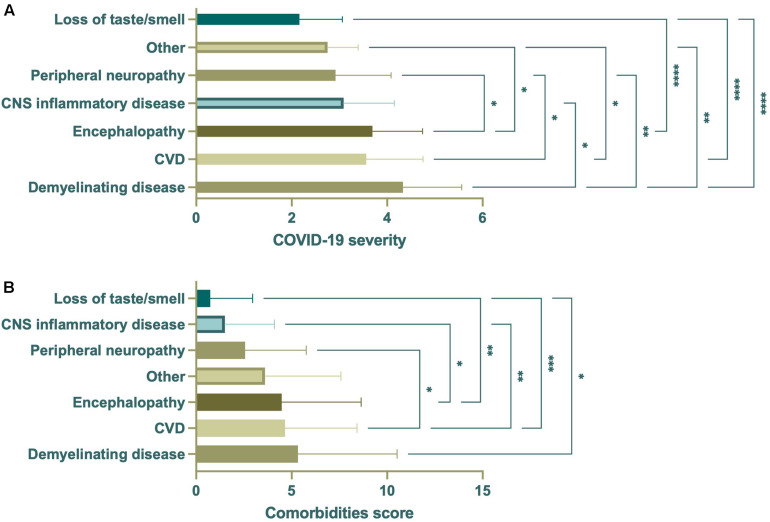
ANOVA of COVID-19 severity or comorbidities score by neurological disease category (*n* = 350). Significant differences in mean COVID-19 severity **(A)** were seen among all neurological disease categories. Demyelinating disease had the highest mean, with CVD and encephalopathy second and third. Similarly, demyelinating disease had the highest mean comorbidities score, with CVD and encephalopathy second and third **(B)**. Loss of taste/smell had the lowest mean COVID-19 severity and comorbidities score. Data were derived from the same subjects. *p < 0.05; **p < 0.01; ***p < 0.001; ****p < 0.0001.

In addition to age, COVID-19 severity and comorbidities appeared to associate with the observance of specific neurological disease (Figure 7). Multivariate analysis of covariance (MANCOVA) to assess if there were significant differences in the linear combination of COVID-19 severity and comorbidities score between the levels of neurological disease category after controlling for age were attempted, however, these tests failed assumptions of homogeneity of covariance and covariate-independent variable independence. As such, ANOVAs were performed separately to assess whether there were significant differences in COVID-19 severity or comorbidities score by neurological disease category. This demonstrated significant differences in the mean COVID-19 severity and comorbidities score among the different neurological disease categories (Figure 8). Demyelinating disease, CVD, and encephalopathy had the highest mean COVID-19 severity and comorbidities score, while loss of taste/smell had the lowest for both, demonstrating a relationship between disease severity and comorbid conditions. To aid viewing, neuronal disease categories were reordered by increasing mean of the two variables (Figure 8).

## Discussion

Neurological manifestations are a significant complication of SARS-CoV-2 infection and COVID-19. Although many anecdotal and case study reports have suggested relationships between neurological complications of disease with age, disease severity, and comorbid conditions, significant associations among these variables remain unclear. Through a systematic review of peer-reviewed, published patient reports spanning the entirety of 2020 through April 4, 2021, and meta-analyses, we report that while smell and/or taste disorders are the most common neurological manifestation of SARS-CoV-2 infection, CVD, manifesting almost entirely as stroke, is a major neurological complication of infection, affecting just over a quarter of individuals in this study. Other clinically significant CNS complications, broadly categorized as encephalopathy, CNS inflammatory disease, demyelinating disease, and peripheral neuropathy have been reported less frequently. Other symptoms, including headache, seizure, aphasia, and ataxia have also been reported in connection with infection without identification of the underlying cause and are categorized as “other” in this report.

When investigating a potential relationship between the type of neurological disorder and age, smell and/or taste disorders remained the most common neurological complication affecting infected individuals 50 years of age and younger. For infected individuals over 50, however, CVD became the most common neurological injury, where it was observed in over half of individuals in this age group. Linear regression analysis, however, suggests only 6% of the variance in CVD is explainable by age. Known risk factors for vascular disease, HTN and DM, as well as critical COVID-19, showed a positive correlation with CVD but with a small effect size. Additionally, stroke affected individuals across the lifespan, including individuals with reported no comorbidities and/or asymptomatic disease. Together, this suggests other factors, which may include virus and/or the host’s response to infection, contribute to the development of CVD.

Although there is currently no clear indicator as to which patients will suffer stroke, several risk factors for stroke in aged individuals have been reported in COVID-19 patients, such as coagulopathy, elevated D-dimer levels, and vascular endothelial dysfunction. A large retrospective study evaluating risk factors for mortality of COVID-19 patients found coagulopathy to be a significant indicator, affecting ∼50% of non-survivors (Zhou et al., 2020). Additionally, marked elevation (<0.5 μg/L) of D-dimer, a by-product of blood clotting that is often elevated in response to acute vascular disease, has been reported in COVID-19 patients and found to be predictive of severe disease and mortality (Gao et al., 2020; Han et al., 2020; Huang et al., 2020; Mao et al., 2020; Zhou et al., 2020).

Endothelial cell infection and/or injury may also contribute to increased risk for CVD with COVID-19. ACE2, the principal receptor used for viral entry, is reportedly expressed by endothelial cells throughout the body, including brain (Hamming et al., 2004; To and Lo, 2004), indicating the potential for viral infection in the endothelium in the CNS. In support of this notion, a post-mortem investigation reported the presence of endothelial cell infection and endotheliitis across the vascular beds of several organs (Varga et al., 2020). Although the brain was not evaluated in this study, the presence of endothelial infection of multiple organs reveals the potential for widespread disruption of vascular homeostasis, increasing the susceptibility of infected patients to CVD. Interestingly, endotheliopathy without evidence of infection has been reported in a cohort of COVID-19 patients (mean age = 62 years), which was associated with severe disease (Goshua et al., 2020). This suggests that the host response to infection may sufficiently promote endothelial cell inflammation and injury, without direct involvement of the virus.

In agreement with endotheliopathy in the CNS, several autopsy and neuroimaging reports demonstrate the presence of brain microvascular lesions and microhemorrhages in COVID-19 patients (Conklin et al., 2020; Fitsiori et al., 2020; Kremer et al., 2020b; Lee et al., 2020; Lin et al., 2020; Radmanesh et al., 2020; Shoskes et al., 2020). Autopsy findings reveal intact endothelium, suggesting that microbleeds may form due to inflammation of endothelial cells that allows for extravasation of red blood cells into the brain parenchyma (Goshua et al., 2020; Pugin et al., 2020). Cerebral microhemorrhages are associated with age and systemic disease, increasing the risk for microhemorrhage development in older patients with COVID-19. Moreover, the integrity of the blood-brain barrier (BBB) decreases with age and is posited to precede and contribute to the development of CVD (Li et al., 2018). The mechanisms of BBB dysfunction in aging are not completely clear; however, small atheromatous plaques, HTN, and endothelial cell inflammation are believed to play a prominent role and may help explain why individuals with underlying comorbidities, including DM and HTN, appear to be at greater risk for developing more severe COVID-19. Additionally, chronic, subclinical inflammation is a common feature of aging that increases the susceptibility of individuals to age-related disease (Franceschi et al., 2000). Chronic inflammation can induce cellular stress and injury that weakens tissues and reduces the ability of cells to counter additional insults. It is reasonable, therefore, that aging-associated inflammation promotes endotheliitis and endotheliopathy, leading to increased “leakiness” of the vasculature that is made more severe with COVID-19.

In addition to CVD, more frequent observations of clinically significant encephalopathy and peripheral neuropathy are also seen in patients over 50 years. Patients with encephalopathy, which is broadly characterized as disease or damage to the brain that affects brain function, present with altered mental status ranging from mild confusion to more severe dementia or coma. Several case reports detail infected patients presenting with acute encephalopathic episodes, irrespective of COVID-19 severity, including acute necrotizing encephalopathy and posterior reversible encephalopathy syndrome (PRES). Encephalopathy accounted for only 5.3% of the total population of COVID-19 patients with neurological manifestations and 10.4% of those over 50 years of age. It is highly probable, however, that due to the strong inflammatory response to infection in the periphery, which can negatively impact the CNS, encephalopathy among infected individuals occurs more frequently but not widely reported in case studies.

Peripheral neuropathy, including Guillain-Barré syndrome (GBS) and critical illness neuromyopathy, have emerged as one of the more serious neurological complications of COVID-19 infection. GBS is a neuromuscular disorder defined as an acute paralytic neuropathy, often preceded by an infection, and clinically characterized by symmetric weakness of the limbs (Yuki and Hartung, 2012). This disease is considered, primarily, to be an affliction of the peripheral nerves that has a 5% fatality rate and results in the severe disability of up 20% of GBS patients (Hughes et al., 2007; Yuki and Hartung, 2012). Critical illness neuromyopathy is characterized by muscle wasting and paralysis and often culminates into a severely disabling weakness of the muscles and/or paralysis (Latronico et al., 2007; Guarneri et al., 2008). In this review, we found that peripheral neuropathy was most frequent among older adults (6.6%, *n* = 44), with zero cases of critical illness neuromyopathy reported in patients younger than 60 years of age.

Less frequent observations of CNS inflammatory disease, demyelinating disease, and smell and/or taste disorders was seen among older adults, as compared to younger adults and patients under 19 years of age (children). CNS inflammatory disorders, which includes encephalitis, myelitis, and meningitis, was most frequently reported among patients under 19 years, while demyelinating disease was similar in frequency among the three age categories. Interestingly, the demyelinating disease, acute disseminated encephalomyelitis (ADEM), which is a rare but serious complication of viral infection most commonly affecting young children, was seen more frequently in older adults (72.8%, *n* = 8), as compared to younger adults (18.1%, *n* = 2) and children (9.1%, *n* = 1), in this review. Impaired smell and/or taste was seen at a high frequency in all age groupings but was the principal manifestation affecting children and young adults and the second most common complication among individuals over 50 years.

### Pathophysiology of Neurological Involvement

**FIGURE 9:**
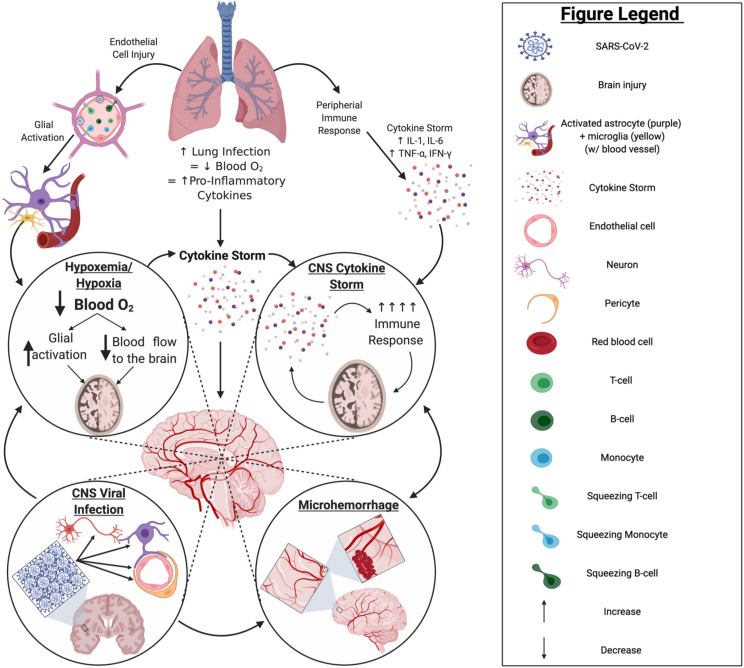
COVID-19 pathology in the CNS: primary impacts and potential mechanisms. Infection by SARS-CoV-2 primarily impacts the lungs, often leading to a hypoxic state and resulting in a robust increase in proinflammatory cytokine production. This may ultimately cause a “cytokine storm” and/or activate glia in the brain. Should such events occur, there is potential for hypoxemia or hypoxia, exacerbated “cytokine storm,” and/or infiltration of peripheral immune cells through endothelial cell infection to occur within the brain; any of which can result in significant brain injury to the infected patient. Although currently unclear, if SARS-CoV-2 can establish a productive or even non-productive infection within the CNS compartment, persistent inflammation and/or impaired cell function may result, increasing the potential for serious injury of the brain and/or pathological brain aging. Created with BioRender.com.

How SARS-CoV-2 infection promotes the development of neurological complications is unclear and may involve several factors (Figure 9). Brain autopsy and CSF analyses seldom report detectable virus in the CNS compartment, however, the neuroinvasive character of other huCoVs suggest SARS-CoV-2 may also infect the CNS (Arbour et al., 2000) and has been demonstrated in a limited number of infected individuals (Filatov et al., 2020; Moriguchi et al., 2020; Poyiadji et al., 2020; Schurink et al., 2020). While the presence of virus in the CNS compartment and mechanism of entry is not fully elucidated, it is highly likely that SARS-CoV-2 is able to gain access to the brain through nasal epithelial cells. ACE2 is highly expressed in nasal epithelium, pointing to the olfactory bulb as a probable point of entry (Sungnak et al., 2020). Infected olfactory epithelial cells may then transfer virus to closely situated olfactory neurons, allowing for retrograde axonal transport into the CNS compartment. In support of this, unilateral oblation of the olfactory bulb prior to intranasal inoculation of a neurotropic coronavirus prevented CNS entry and viral spread in mouse brain (Perlman et al., 1990).

Hematological entry of virus into the CNS also cannot be ruled out. SARS-CoV-2 has been detected in endothelial cells throughout the body of infected subjects and, given the prevalence of endothelial ACE2 expression, SARS-CoV-2 may be found in brain endothelium (Hamming et al., 2004; Varga et al., 2020). Post-mortem analyses of human and non-human primate brain revealed hCoV-299E in brain endothelial cells (Cabirac et al., 1995). Viral infection of the endothelial cells by coronavirus has been found to cause inflammation of the endothelial cells which disrupts vascular homeostasis and coagulation, suggesting an increased risk for CVD, as a result (Cabirac et al., 1995; Varga et al., 2020).

Even in the absence of direct neuronal or neural cell infection, hypoxia/hypoxemia, coagulopathy, and uncontrolled inflammation or “cytokine storm” can also negatively impact the CNS and cognition (Figure 9). Indeed, most clinical evidence suggests neurological complications of COVID-19 are due to secondary effects of infection, including reduced O_2_ and hyperimmune responses, often referred to as “cytokine storm.” Serum levels of pro-inflammatory cytokines [e.g., interleukin (IL)-6, IL-8, tumor necrosis factor-α (TNF-α)] in COVID-19 patients are significantly predictive and/or correlative to the severity of infection and mortality (Chen T. et al., 2020; Gao et al., 2020; Huang et al., 2020; Zhou et al., 2020). Previously, SARS-CoV patients with severe disease were found to have elevated levels of pro-inflammatory cytokines and chemokines, and reduced levels of anti-inflammatory cytokines (IL-10), in comparison to patients with mild disease (Chien et al., 2006). Indeed, virus-associated diseases of the nervous system, such as acute necrotizing encephalopathy, are associated with high levels of pro-inflammatory cytokines in serum and CSF. As such, elevated pro-inflammatory cytokines in serum of severe COVID-19 patients may promote inflammation in brain and contribute to the neurological manifestations of disease (Kansagra and Gallentine, 2011; Sun et al., 2019).

### Long-Term Impact of COVID-19 on the Nervous System

The long-term consequences of COVID-19-associated nervous system injury and/or dysfunction is currently unknown, however, reports continue to emerge describing persistent symptoms of disease months after resolution of infection, including impaired smell and/or taste, chronic fatigue, and impaired cognition. Long-term complications of infection are referred to as post-acute sequelae of COVID-19 (PASC) or Long COVID and evidence for this complication is seen with other viruses that induce neurological disease, including human immunodeficiency virus (HIV), West Nile virus, and multiple herpes- and picornaviruses. It is not entirely clear if SARS-CoV-2 directly infects neurons and/or non-neuronal cells of the CNS and, if so, whether virus is eradicated from these sites with recovery of COVID-19. The significance of this important consideration is seen in a single case report of a 78-year-old woman who recovered from COVID-19 but succumbed to a sudden cardiac arrest prior to hospital discharge (Yao et al., 2020). Although this individual had three consecutive SARS-CoV-2 PCR negative nasopharyngeal swabs, postmortem investigation of multiple tissues, excluding brain, revealed residual virus in lung (Yao et al., 2020). These findings suggest that the virus may not be completely cleared in some patients that appear to have recovered and raises the possibility that SARS-CoV-2 may evade immune surveillance, at least to some degree. It is important to note that replication-competency of virus found in lung of this patient was not determined and remains an important scientific and clinical question. This would have major implications for the brain if replication-competent virus persists in the CNS compartment after recovery. Even an abortive infection, if present, could negatively impact cell function and impair brain homeostasis. Alternatively, or in addition to direct viral involvement, chronic neuroinflammation can contribute to impaired brain homeostasis through production of soluble factors that directly and/or indirectly impair neuronal function. Clinical follow-up and prospective observational studies are critical for assessing long-term neurological outcomes of patients recovered from COVID-19. While this is a likely standard for follow-up of individuals who were diagnosed with serious neurological manifestations, functional and cognitive decline may continue after recovery among COVID-19 patients for whom neurological disease was not identified. There is significant evidence that supports the notion that these individuals, particularly those recovered from severe COVID-19, may have difficulty performing critical functions long after recovery, including reduced job performance and/or ability to attend to activities of daily living, that may worsen over time.

Limited studies are available that investigate the long-term neurological consequences of COVID-19 and do not include neurological assessments but have relied on neuropsychological testing and self-reports. A large cross-sectional study involving cognitive assessments of subjects who had recovered from COVID-19 demonstrated impairment in a variety of cognitive domains and a lower global cognitive performance score, with worsening performance associated with the severity of respiratory disease (Hampshire et al., 2020). This may suggest irreversible injury to the brain due to reduced oxygen and/or chronic subclinical unresolved neuroinflammation, two factors that play a major role in pathologic brain aging. Importantly, minor deficits were even seen among individuals who had experienced only mild respiratory symptoms. Additional follow-up of these individuals is needed to assess the course of symptoms with increased recovery time. It is important to note that testing was performed on-line, rather than by a board-certified neuropsychologist. In a separate assessment of self-reports from subjects at 6-month post-infection, sleep difficulties, anxiety, and depression were the most commonly reported complaints, in addition to fatigue and muscle weakness (Huang et al., 2021).

Evidence of injury at the level of the CNS is very limited at this time, however, a functional imaging study of patients with persistent anosmia following recovery of SARS-CoV-2 infection displayed reduced metabolism in bilateral limbic cortices and the insular cortex of the left hemisphere, as compared to controls (Donegani et al., 2021). This suggests brain involvement in SARS-CoV-2-associated anosmia that may also impact cognitive function, as these brain regions are involved in multiple cognitive processes, including learning and memory, word, face, and body recognition, and consciousness. The insular cortices are also involved in taste, which is often impaired in SARS-CoV-2 infection, alone or concurrent with impaired smell, which may implicate injury within in this region among individuals suffering loss of taste and/or smell. With the potential for controlling the SARS-CoV-2 pandemic with the world-wide introduction of multiple vaccines, more comprehensive follow-up of recovered patients is likely to become an urgent public health concern, including neurological work-up and neuropsychological and/or psychiatric assessments.

This systematic review of the literature and meta-analysis has demonstrated that neurological manifestations are a common complication of SARS-CoV-2 infection and COVID-19 that effects individuals across the lifespan, with all severities of COVID-19, and with or without comorbidities. Consistently emerging case reports and retrospective studies detailing the neurological impact of COVID-19, point to the necessity for investigating the impact of hyperimmune responses and/or reduced oxygen more thoroughly on neuronal injury. In addition, the neuroinvasive potential of the virus should not be ruled outs, as neurological conditions may be seen as a presenting symptom of infection and arise in the absence of respiratory disease. Further, as SARS-CoV-2 infection can lead to devastating neurological diseases irrespective of age, sex, or comorbidities, targeted studies of the COVID-19 population are imperative to better understand and elucidate the true impact of COVID-19 on the CNS. COVID-19 patients need to be followed for potential long-term neurological sequelae after recovery from infection, including pathologic brain aging, that likely plays a key role in PASC. With multiple vaccines now available, we may continue to see a reduction in new cases and/or disease severity, however, the potential for CNS complications remains a major clinical and public health concern.

## Author Contributions

BS and TF contributed to the writing of the article, figure and table preparation, and final assembly. Both authors contributed to the article and approved the submitted version.

## Conflict of Interest

The authors declare that the research was conducted in the absence of any commercial or financial relationships that could be construed as a potential conflict of interest.

## Publisher’s Note

All claims expressed in this article are solely those of the authors and do not necessarily represent those of their affiliated organizations, or those of the publisher, the editors and the reviewers. Any product that may be evaluated in this article, or claim that may be made by its manufacturer, is not guaranteed or endorsed by the publisher.

## Footnotes

1
https://analyze.intellectusstatistics.com
