# Supplementary material for: Age-Associated Neurological Complications of COVID-19: A Systematic Review and Meta-Analysis
Source: Front Aging Neurosci. 2021 Aug 2;13:653694. doi: 10.3389/fnagi.2021.653694 (PMC8366271; doi:10.3389/fnagi.2021.653694)
Supplement: Supplementary file 1 [file Table_1.pdf]

Supplementary Table 1. Frequencies and percentages of specific diagnoses included under each category of neurological complications.

**Category**<br>Diagnosis	*n*	%
**Cerebrovascular disease**	**259**	
acute ischemic stroke	1	0.39
acute peripheral artery occlusion and ischemic stroke	1	0.39
bilateral occipital stroke w/loss of vision	1	0.39
CVST, thrombotic stroke	3	1.17
CVT	2	0.78
diffuse microvascular occlusion	4	1.56
embolic stroke	5	1.95
hemorrhage	12	4.67
hemorrhages, encephalopathy	2	0.78
hemorrhages, microhemorrhages	1	0.39
hemorrhagic PRES	2	0.78
hemorrhagic stroke	3	1.17
intraparenchymal hemorrhage	2	0.78
ischemic stroke	16	6.23
ischemic stroke with embolic infarcts	1	0.39
ischemic stroke with large vessel occlusion	1	0.39
large hemorrhage CVT, status epilepticus, encephalopathy	1	0.39
large vessel stroke	6	2.33
microhemorrhage	3	1.17
microhemorrhage, hypoxic injury, edema	10	3.89
microhemorrhage, lesion	1	0.39
microhemorrhages, encephalopathy	1	0.39
microhemorrhages, hypoxic injury, delirium	2	0.78
microhemorrhages, hypoxic injury, delirium, aguesia	1	0.39
microhemorrhages, lesions	1	0.39
multiple embolic strokes, encephalopathy	1	0.39
reversible cerebral vasoconstriction syndrome	1	0.39
SAH	4	1.56
SAH, encephalopathy	2	0.78
stroke	154	60.31
stroke, Balint-Holmes' syndrome, anosmia, ageusia	1	0.39
stroke, microhemorrhage, encephalopathy	1	0.39
stroke, microhemorrhages	1	0.39
stroke, seizures, encephalopathy	1	0.39
thrombotic stroke	6	2.33
vasculitis-related stroke	1	0.39
**CNS inflammatory disease**	**48**	
acute hemorrhagic leukoencephalomyelitis	1	2.13
acute hemorrhagic necrotizing encephalitis	2	4.26
acute transverse myelitis, anosmia, dysgeusia	1	2.13

anti-NMDAR encephalitis	2	4.26
CLOCCs	3	6.38
encephalitis	2	4.26
encephalitis and myelitis	1	2.13
encephalitis with seizure	2	4.26
encephalitis with seizure, aphasia	1	2.13
encephalitis, vasculitis	1	2.13
endotheliitis	1	2.13
meningoencephalitis	22	46.81
myelitis	5	10.64
reversible lesion of the corpus callosum	2	4.26
viral cerebellitis	1	2.13
**Demyelinating disease**	**15**	
acute demyelination	1	6.67
acute multifocal demyelinating lesions	1	6.67
acute tumefactive demyelination	1	6.67
ADEM	9	60.00
ADEM w/pseudoleukodystrophy	1	6.67
ADEM, GBS	1	6.67
demyelinating polyradiculoneuritis with GBS	1	6.67
**Encephalopathy**	**58**	
acute necrotizing encephalopathy	2	3.45
altered mental status	1	1.72
aphasia, behavioural agitation	1	1.72
delirium	3	5.17
disorientation, extreme fatigue, disgeusia	1	1.72
encephalopathy	31	53.45
encephalopathy with aphasia	1	1.72
encephalopathy with reversible splenium lesion	1	1.72
encephalopathy, aguesia	1	1.72
encephalopathy, hyposmia	1	1.72
hyponatremic encephalopathy	1	1.72
hypoxic-ischemic encephalopathy	3	5.17
impaired consciousness, ataxia	1	1.72
impaired consciousness, headache	1	1.72
impaired consciousness, wm lesions	1	1.72
opthalmoparesis, encephalopathy	1	1.72
opthalmoparesis, encephalopathy, right hemicranial headache	1	1.72
periventricular and deep wm injury	1	1.72
PRES	4	8.62
**Peripheral neuropathy**	**64**	
acute motor axonal neuropathy	1	1.59
AMSAN GBS	5	7.94
anosmia, ageusia, GBS	2	3.17
critical illness neuromyopathy	5	7.94
dysgeusia, GBS	1	1.59

facial nerve palsy, anosmia	1	1.59
GBS	33	52.38
GBS and Bell's Palsy	1	1.59
GBS with dysautonomia	1	1.59
Miller Fisher GBS	3	4.76
Miller Fisher GBS, ataxia	1	1.59
Miller Fisher GBS, ataxia, polyneuritis cranialis	2	3.17
ophthalmoparesis, facial palsy	2	3.17
polyneuritis cranialis GBS	1	1.59
polyneuritis cranialis GBS, hyposmia, ageusia	4	6.35
**Smell and/or taste disorders**	**37**	
ageusia	5	13.89
anosmia	11	30.56
anosmia, ageusia	10	27.78
anosmia, ageusia, headache	3	8.33
anosmia, ageusia, hearing impairment, lethargy, headache	1	2.78
anosmia, dysguesia, olfactory bulb edema	1	2.78
anosmia, headache	2	5.56
anosmia, hypogeusia, akinetic-rigid parkinsonism	1	2.78
anosmia, olfactory bulb atrophy	1	2.78
hypoguesia	1	2.78
**Other**	**37**	
cerebral edema	1	2.78
chorioretinopathy, Adie syndrome, headache	1	2.78
headache	2	5.56
headache with photophobia	1	2.78
intracranial hypertension (headache)	13	36.11
moderate neck stiffness, photophobia, somnolence	1	2.78
myoclonus	8	22.22
non-remitting headache	1	2.78
seizure	7	19.44
severe brain edema w/seizure	1	2.78

Note. Due to rounding errors, percentages may not equal 100%.

Abbreviations: Cerebral venous sinus thrombosis (CVST); Cerebral venous thrombosis (CVT); Posterior reversible encephalopathy syndrome (PRES); Subarachnoid hemorrhage (SAH); Central nervous system (CNS); N-Methyl-D-aspartate receptor (NMDAR); Cytotoxic lesions of corpus callosum (CLOCC); Acute disseminated encephalomyelitis (ADEM); Guillain-Barré syndrome (GBS); Acute motor-sensory axonal neuropathy (AMSAN)
