# Supplementary material for: Age-Associated Neurological Complications of COVID-19: A Systematic Review and Meta-Analysis
Source: Front Aging Neurosci. 2021 Aug 2;13:653694. doi: 10.3389/fnagi.2021.653694 (PMC8366271; doi:10.3389/fnagi.2021.653694)
Supplement: Supplementary file 2 [file Table_2.pdf]

Supplementary Table 2. Frequencies and percentages of all reported comorbidities

	n	%
Comorbidities		
AKI, obesity	1	0.28
aplastic anemia	1	0.28
asthma	1	0.28
breast cancer	2	0.55
Crohn's disease, obesity	1	0.28
DM	21	5.79
DM, asthma	1	0.28
DM, hyperlipidemia	4	1.10
DM, hyperthyroidism	1	0.28
DM, obesity	1	0.28
DM, CAD	1	0.28
dyslipidemia	2	0.55
dyslipidemia, atrial fibrilation	1	0.28
HTN	55	15.15
HTN, asthma	3	0.83
HTN, atrial fibrilation, smoker	1	0.28
HTN, CKD, smoker	1	0.28
HTN, dyslipidema	4	1.10
HTN, dyslipidema, CKD	1	0.28
HTN, dyslipidemia, atrial fibrilation	1	0.28
HTN, heart failure	1	0.28
HTN, hypercholesterolemia	4	1.10
HTN, hyperlipidemia	8	2.20
HTN, hyperlipidemia, asthma	1	0.28
HTN, hyperlipidemia, CAD	1	0.28
HTN, hyperlipidemia, CHF	1	0.28
HTN, kidney failure	1	0.28
HTN, KTR, past smoker	1	0.28
HTN, migraine	2	0.55
HTN, obesity	5	1.38
HTN, obesity, CKD	1	0.28
HTN, obesity, dyslipidemia, sleep apnea, ESRD	1	0.28
HTN, obesity, hyperlipidemia	1	0.28
HTN, smoking	5	1.38
HTN, CAD	3	0.83
HTN, CKD	4	1.10
HTN, DM	29	7.99
HTN, DM, CAD	1	0.28
HTN, DM, cholithiasis	1	0.28
HTN, DM, CKD	4	1.10
HTN, DM, dyslipidemia	3	0.83
HTN, DM, dyslipidemia, ESRD, smoker, sleep apnea	1	0.28

HTN, DM, ESRD	1	0.28
HTN, DM, hyperlipidemia	6	1.65
HTN, DM, hyperlipidemia, CAD	2	0.55
HTN, DM, hyperlipidemia, CAD, CHF	1	0.28
HTN, DM, ischemic heart disease	1	0.28
HTN, DM, obesity	5	1.38
HTN, DM, obesity, AKI	1	0.28
HTN, DM, obesity, CHD	1	0.28
HTN, DM, obesity, hyperlipidemia, CKD	1	0.28
HTN, DM, obesity, smoker	1	0.28
hyperlipidemia	3	0.83
hyperthyroidism	1	0.28
ischemic heart disease	1	0.28
left eye strabismus	1	0.28
migraine	2	0.55
none	131	36.09
obesity	10	2.75
obesity, asthma	3	0.83
obesity, smoking	1	0.28
psoriatic arthritis	1	0.28
RA, allergic alveolitis, monoclonal IgG kappa gammopathy	1	0.28
sickle cell disease	1	0.28
sinusitis	1	0.28
sleep apnea	1	0.28
smoking	1	0.28
ulcer	1	0.28

Note. Due to rounding errors, percentages may not equal 100%.

Abbreviations: Acute kidney injury (AKI); Diabetes mellitus (DM); Coronary artery disease (CAD); Chronic kidney disease (CKD); Coronary heart failure (CHF); Kidney transplant recipient (KTR); End-stage renal disease (ESRD); Rheumatoid arthritis (RA)
