# Supplementary material for: Age-Associated Neurological Complications of COVID-19: A Systematic Review and Meta-Analysis
Source: Front Aging Neurosci. 2021 Aug 2;13:653694. doi: 10.3389/fnagi.2021.653694 (PMC8366271; doi:10.3389/fnagi.2021.653694)
Supplement: Supplementary file 3 [file Table_3.pdf]

Supplementary Table 3. Large study patient reports of COVID-19 from which individual patient data could not be collected (n = 1689).

**First Author** [reference]	Neurological Diagnosis	Age (years)	Sex	COVID-19 severity	Comorbidities	*n*
**Cagnazzo**<br>[282]	Stroke	71 [59–79]<br>Median [IQR]	61 Male<br>30 Female<br>2 non-disclosed	Asymptomatic (n=32)<br>Moderate-severe (n=61)	Hypertension (n=62)<br>Diabetes (n=20)<br>Hypercholesterolemia (n=28)<br>Smoking (n=21)<br>Kidney disease (n=4)	93
**Ji**<br>[283]	Stroke	66.4 ± 12.1<br><br>**<60 years**<br>51.9 ± 5.4<br><br>**≥ 60 years**<br>72.5 ± 8.3<br><br>**Non-severe COVID-19**<br>62.4 ± 12.0<br><br>**Severe COVID-19**<br>74.3 ± 8.0<br>Mean ± SD	**<60 years**<br>3 Male<br>5 Female<br><br>**≥ 60 years**<br>12 Male<br>7 Female<br><br>**Non-severe COVID-19**<br>7 Male<br>11 Female<br><br>**Severe COVID-19**<br>3 Male<br>6 Female	Non-severe (n=18)<br>Severe (n=9)	**<60 years**<br>Hypertension (n=3)<br>Diabetes (n=2)<br>History of smoking (n=1)<br><br>**≥ 60 years**<br>Hypertension (n=10)<br>Diabetes (n=5)<br>Cardiovascular disease (n=2)<br>History of smoking (n=7)<br><br>**Non-severe COVID-19**<br>Hypertension (n=5)<br>Diabetes (n=5)<br>History of smoking (n=6)<br><br>**Severe COVID-19**<br>Hypertension (n=8)<br>Diabetes (n=2)<br>Cardiovascular disease (n=2)<br>History of smoking (n=2)	27
**Kihira**<br>[284]	Stroke	65.6 ± 13.2<br>Mean ± SD	77 Male<br>49 Female	Data not provided	Cardiovascular risk factors* (n=218)<br>Atrial Fibrillation (n=19)<br>Pulmonary diseases^ (n=41)<br>Smoking (n=41)	126
**Lechien**<br>[285]	Smell disorder (n=357)<br>Taste disorder (n=342)	36.9 ± 11.4<br>[19-77]<br>Mean ± SD [range]	154 Male<br>263 Female	Mild-moderate	Smoking (n=56)<br>Allergies (n=85)	417
**Mowla**<br>[286]	Cerebral venous sinus thrombosis	50.9 ± 11.2	5 Male<br>8 Female	Asymptomatic (n=1)<br>Mild-moderate (n=9)<br>Severe (n=1)	Not provided	13

**Porta-Etessam** [287]	Headache	43.4 ± 11.4<br>mean ± SD	21 Male<br>91 Female	Mild-moderate	Hypertension (n=5)<br>Dyslipidemia (n=15)	112
**Qiu** [288]	**Children**<br>Smell disorder (n=3)<br>Smell & taste disorder (n=7)<br><br>**Adults**<br>Smell disorder (n=61)<br>Taste disorder (n=7)<br>Smell & taste disorder (n=93)	**Children**<br>16.6 ± 0.7<br>[16.3-17]<br><br>**Adults**<br>38.8 ± 17.6<br>[23-53]<br>Median ± SD [IQR]	**Children**<br>6 Male<br>4 Female<br><br>**Adults**<br>92 Male<br>69 Female	**Children**<br>Mild (n=6)<br>Moderate (n=4)<br><br>**Adults**<br>Mild (n=76)<br>Moderate (n=40)<br>Severe (n=33)<br>Critical (n=10)	Not provided	171
**Scullen**[a] [200]	Encephalopathy (n=20)<br>Acute necrotizing Encephalopathy (n=2)<br>Vasculopathy (n=5)	59.8 [35-91]<br>Mean [range]	14 Male<br>13 Female	Critical	Hypertension (n=17)<br>Diabetes (n=14)<br>Obesity (n=7)<br>Kidney disease (n=6)<br>Cardiovascular disease (n=7)	27
**Trifan**[b] [80]	Stroke	64 [18]<br><br>**Male**<br>63 [17]<br><br>**Female**<br>68 [17]<br>Median [IQR]	44 Male<br><br>39 Female	**Male**<br>Asymptomatic (n=3)<br>Mild-moderate (n=9)<br>Severe (n=32)<br>Critical (n=27)<br><br>**Female**<br>Asymptomatic (n=3)<br>Mild-moderate (n=17)<br>Severe (n=19)<br>Critical (n=16)	**Male**<br>Cardiovascular risk factors* (n=83)<br>Pulmonary Diseases^ (n=17)<br>Atrial fibrillation (n=5)<br>Kidney disease (n=10)<br>Chronic Obstructive Pulmonary Disease or Asthma (n=2)<br><br>**Female**<br>Cardiovascular risk factors* (n=59)<br>Pulmonary diseases^ (n=10)<br>Atrial fibrillation (n=3)<br>Kidney disease (n=12)<br>Chronic Obstructive Pulmonary Disease or Asthma (n=2)	83
**Uginet** [289]	Encephalopathy	64.6 ± 12.1<br><br>**Mild COVID-19**<br>63.8 ± 15.0	**Mild COVID-19**<br>17 Male	Mild (n=17)<br>Severe (n=14)	**Mild COVID-19**<br>Smoking (n=1)<br>Cardiovascular risk factors* (n=12)	31

		**Severe COVID-19**<br>65.6 ± 7.8<br>Mean ± SD	**Severe COVID-19**<br>12 Male<br>2 Female		Chronic cardiac disease (n=4)<br>Pulmonary diseases^ (n=4)<br>Dementia (n=3)<br><br>**Severe COVID-19**<br>Smoking (n=2)<br>Cardiovascular risk factors* (n=10)<br>Chronic cardiac disease (n=4)<br>Pulmonary diseases^ (n=3)	
**Vaira**<br>[290]	Smell disorder (n=55)<br>Taste disorder (n=76)	49.6 ± 8.5<br>[43–55.2]<br>Mean ± SD<br>[IQR]	53 Male<br>53 Female	Moderate	Not provided	106
**Vaira**<br>[291]	Smell disorder (n=84)<br>Taste disorder (n=56)	51.2 ± 8.8<br>[46.7–58.0]<br>Mean ± SD<br>[IQR]	68 Male<br>70 Female	Mild-moderate (n=106)<br>Severe-critical (n=32)	Diabetes (n=15)<br>Obesity (n=40)<br>Cardiovascular disease (n=37)<br>Pulmonary diseases^ (n=21)	138
**Vaira**<br>[292]	Smell disorder (n=225)<br>Taste disorder (n=234)	48.5 ± 12.8<br>[23-88]<br>Mean ± SD<br>[range]<br><br>**≤50 years**<br>(n=204)<br><br>**>50 years**<br>(n=141)	146 Male<br>199 Female	Asymptomatic (n=10)<br><br>**Smell disorder**<br>Mild (n=122),<br>Moderate (n=77)<br>Severe (n=36)<br><br>**Taste disorder**<br>Mild (n=118)<br>Moderate (n=82)<br>Severe (n=34)	Not provided	345

[a]Data from three case reports given as example were included in the statistical analysis
[b]Data from ten case reports given as example were included in the statistical analysis
*Cardiovascular risk factors: hypertension; diabetes, dyslipidemia, obstructive sleep apnea
^Pulmonary diseases: coronary artery disease or congestive heart failure
Abbreviations: Standard deviation (SD); Interquartile range (IQR)
